# Emerging roles of non-coding RNA derived from extracellular vesicles in regulating PD-1/PD-L1 pathway: insights into cancer immunotherapy and clinical applications

**DOI:** 10.1186/s12935-025-03809-8

**Published:** 2025-05-23

**Authors:** Haixia Zhang, Lianfeng Gong, Li Yu, Chenge Xian, Zhaowu Ma, Xianwang Wang, Ruohan Xia

**Affiliations:** 1 Health Science Center, Yangtze University, Nanhuan Road 1, Jingzhou, 434023 Hubei China; 2 Department of Urology, General Hospital of The Yangtze River Shipping, Wuhan, 430010 China; 3 Naidong District People’s Hospital, Shannan, 856004 Tibet Autonomous Region China; 4 Shannan Maternal and Child Health Hospital, Shannan, 856099 Tibet Autonomous Region China

## Abstract

Numerous studies have demonstrated that extracellular vesicles (EVs) carry a variety of noncoding RNAs (ncRNAs), which can be taken up by neighboring cells or transported to distant sites via bodily fluids, thereby facilitating intercellular communication and regulating multiple cellular functions. Within the tumor microenvironment, EV-ncRNA, on the one hand, regulate the expression of PD-L1, thereby influencing tumor immune evasion, promoting tumor cell proliferation, and enhancing tumor growth, invasion, and metastasis in vivo. On the other hand, these specific EV-ncRNAs can also modulate the functions of immune cells (such as CD8 + T cells, macrophages, and NK cells) through various molecular mechanisms, inducing an immunosuppressive microenvironment and promoting resistance to anti-PD-1 therapy. Therefore, delving into the molecular mechanisms underlying EV-ncRNA regulation of immune checkpoints presents compelling therapeutic prospects for strategies that selectively target EV-ncRNAs. In this review, we elaborate on the cutting-edge research progress related to EV-ncRNAs in the context of cancer and dissect their pivotal roles in the PD-1/PD-L1 immune checkpoint pathway. We also highlight the promising clinical applications of EV-ncRNAs in anti-PD-1/PD-L1 immunotherapy, bridging basic research with practical clinical applications.

**Graphical Abstract:**

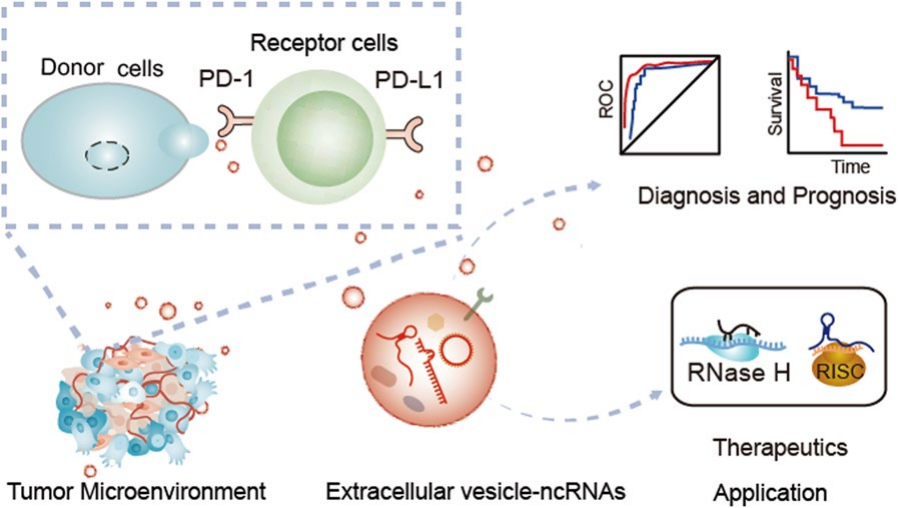

## Introduction

Extracellular vesicles (EVs), which include exosomes, apoptotic bodies and microvesicles (MVs), are small membrane-bound structures that originate from multivesicular bodies or the plasma membrane [1, 2]. Most, if not all, cell types are capable of releasing these vesicles into bodily fluids, and they contain a subset of proteins, lipids, and ncRNAs originating from the parent cell [3, 4]. EVs can be taken up by nearby cells or transported to distant sites through body fluids [5, 6]. This process facilitates intercellular communication and modulates various cellular functions [7, 8]. EVs are pivotal in human physiological and pathological processes, such as intercellular communication, tissue regeneration and repair, immune response, and cancer progression [9–11]. Noncoding RNAs (ncRNAs) are a diverse class of RNA molecules that do not have protein-coding ability [12]. This class includes microRNAs (miRNAs) [13], long noncoding RNAs (lncRNAs), and circular RNAs (circRNAs) [14, 15]. MiRNAs are short RNA molecules, approximately 22 nucleotides in length [16]. As a class of ncRNA transcripts, miRNAs play a crucial role in regulating gene expression by disrupting the translation and/or stability of messenger RNA (mRNA) or by modulating the transcription of target mRNAs [13, 17, 18]. Over the past decade, miRNAs have been recognized as key players in the characteristic manifestations of cancer [19–21]. CircRNAs are RNA molecules that form a closed loop through back-splicing [2]. They have a circular structure with multiple binding sites for miRNAs, referred to as miRNA response elements (MREs) [22, 23]. This unique structure endows circRNAs with a high degree of stability, enabling them to resist degradation by RNA exonucleases and remain within cells [24–26]. Notably, circRNAs have been observed to regulate the expression of immune checkpoint molecules within tumor cells, potentially affecting the therapeutic efficacy of immune checkpoint inhibitors (ICIs) [2]. LncRNAs are described as transcripts longer than 200 nucleotides, lacking any protein-coding ability [27, 28].

Numerous studies indicate that both lncRNAs and circRNAs share overlapping functions.These functions include modulating transcription, processing and stabilizing mRNA, sequestering miRNAs, and interacting with proteins [14–30]. Despite their relatively low abundance, lncRNAs and circRNAs show unique expression patterns in different cell types, tissues, diseases, and developmental stages [31–34]. Additionally, ncRNAs are protected from degradation when they are encapsulated in EVs, which helps maintain their stability and function in the TME [35]. Notably, these EV-ncRNAs can be easily detected in various bodily fluids, such as tissues, serum, plasma, and urine, making them accessible for analysis [36, 37]. The immune system is crucial in controlling cancer progression [38]. Cancer immunotherapy is developed to precisely target tumors by stimulating the immune system and generating strong antitumor responses [39–41]. This immunotherapy strategy relies on identifying immune checkpoints such as CTLA-4, PD-1, and PD-L1, which suppress immune activity [42–44]. Recent studies have found that EV-ncRNAs are involved in the regulation of the PD-1/PD-L1 pathway, which plays a significant role in cancer [45–48]. The introduction of ICIs, particularly successful anti-PD-1 antibodies, has significantly transformed cancer therapy, delivering substantial clinical benefits to some patients with various types of cancer. Nevertheless, many patients continue to face both primary and acquired resistance to these new anti-PD-1 therapies [49].

Therefore, this review consolidates and evaluates recent findings on the functions and regulatory mechanisms of EV-ncRNAs in the PD-1/PD-L1 pathway related to cancer immunotherapy. We emphasize the potential clinical applications of EV-ncRNAs in diagnosis and therapy. Additionally, we explore future research avenues and highlight the significance of understanding the complex interactions between EV-ncRNAs and the immune system in cancer.

## Emerging roles of extracellular vesicles and ncRNAs in cancer biology.

### Biogenesis, release, and uptake of EVs

**Fig. 1:**
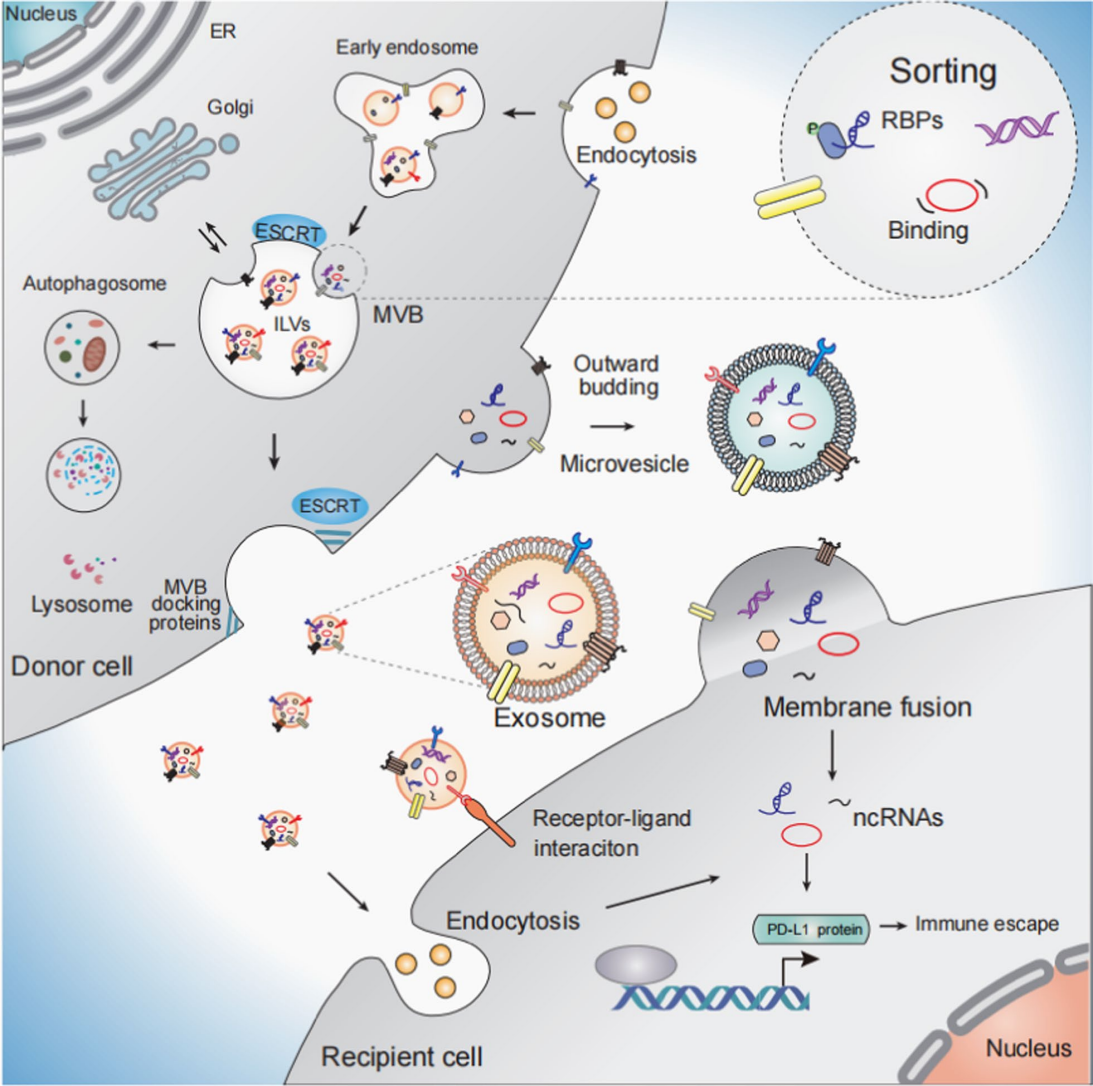
Extracellular vesicle biogenesis, release, and uptake The biogenesis and secretion of extracellular vesicles (EVs) by donor cells, followed by their internalization into target cells via receptor‒ligand interactions, endocytosis, and membrane fusion, ultimately lead to functional impacts within the recipient cells

EVs constitute a heterogeneous group of membrane-enclosed vesicles that arise from either the endosome or the plasma membrane [50]. According to the literature, specific classes of EVs encompass apoptotic bodies, exosomes, and microvesicles (MVs), which exhibit variations in their size, origin, membrane protein expression, and interior cargo [51–54]. Here, we elucidate the processes of biogenesis, secretion, and absorption pertaining to the most extensively investigated exosomes and MVs (Fig. 1). Exosomes are generated via a process involving double invagination of the plasma membrane and the creation of intracellular multivesicular bodies (MVBs) containing intraluminal vesicles (ILVs). These ILVs are ultimately secreted as exosomes, ranging in size from ~ 40 to 160 nm in diameter [55, 56]. Inside MVBs, these ILVs can sequester various biomolecules, including proteins, lipids, and RNAs. Upon their formation, MVBs can either undergo fusion with lysosomes to degrade their cargo or integrate with the plasma membrane, resulting in the release of ILVs into the extracellular space as exosomes [55–57]. Other EVs, such as MVs, are generated through the outward protrusion of the plasma membrane, originating from a direct pinching and budding mechanism [58, 59]. The generation of ILVs within MVBs can proceed via ESCRT (Endosomal Sorting Complex Required for Transport)-dependent or ESCRT-independent pathways [60–62]. The specificity of vesicle formation and release can vary depending on the cellular context, influencing the type of cargo, including ncRNAs, packaged into these vesicles [63, 64].

The selection of ncRNAs into EVs is a highly regulated process, with recent evidence suggesting that specific sequences or motifs within ncRNAs are recognized by RNA-binding proteins such as hnRNPA2B1 and YBX1, which mediate their inclusion into EVs [65–67]. Furthermore, lipid raft-associated proteins such as tetraspanins (CD63, CD81) and ESCRT-related components assist in the loading of select ncRNAs into exosomes [68]. This selective loading allows EVs to carry specific ncRNA cargos, enabling cancer cells to communicate and altering the behavior of distant cells, such as promoting metastasis [68, 69]. EVs are internalized by target cells via multiple pathways, including receptor‒ligand interactions, endocytosis, and membrane fusion. Once internalized, the ncRNAs within EVs can modulate gene expression [55–58]. EVs can be derived from multiple diagnostic media, such as breast milk, saliva, urine and blood [70, 71]. The isolation of EVs, particularly exosomes, presents a technical challenge because of their small size and similarity to other vesicles. Technological advances have enabled the creation of cost-efficient, efficient, and versatile isolation techniques, such as microfluidics, thermophoresis, and magnetic separation [72]. Immunoaffinity-based methods targeting specific exosomal markers, such as CD9, CD63, and CD81, have also been used to increase the specificity of isolation [73]. However, emerging techniques such as microfluidics and nanoplasmonic sensors are improving the efficiency and purity of exosome isolation, which is crucial for downstream applications in cancer diagnostics and therapeutics [70–76].

### Dual roles of EV-ncRNAs in tumor progression and immune modulation

EVs are crucial mediators of intracellular communication among stroma, immune, and tumor cells [77, 78]. They transport bioactive molecules, including RNA, DNA, proteins, and lipids, however, the precise role of these molecules remains largely elusive [79, 80]. Given the advancements in gene sequencing technology, the cellular cargo within EVs can now be characterized with greater precision, facilitating more extensive mechanistic studies [81–84].

In recent years, increasing evidence has elucidated the critical roles and potential applications of EV-ncRNAs in cancer progression. EV-ncRNAs, which resemble a double-edged sword, play roles in tumor metastasis, angiogenesis, immunosuppression, and the formation of a premetastatic microenvironment [77–85]. For example, dysregulated EV-ncRNAs influence the expression of molecules associated with epithelial‒mesenchymal transition (EMT) and angiogenesis by interacting with relevant signaling pathways in recipient cells [86, 87]. EV-miR-103a derived from hypoxic lung cancer cells increase M2-type macrophage polarization, leading to decreased PTEN levels and subsequent enhanced activation of AKT and STAT3, as well as increased expression of immunosuppressive and pro-angiogenic factors. Furthermore, inhibition of miR-103a with an miRNA inhibitor effectively reduced hypoxic cancer-mediated M2-type polarization and improved the cytokine profile of tumor-infiltrating macrophages [88]. Additionally, exosomal PD-L1 can induce apoptosis in activated T cells, further inhibiting immune responses [81]. Conversely, certain EV-RNAs have been identified as key inhibitors that promote tumor cell apoptosis and inhibit tumor immune evasion [89]. For example, two miRNAs endogenously expressed in macrophages, namely miR-142 and miR-223, play crucial roles in inhibiting the proliferation of hepatocellular carcinoma (HCC) [90]. In the context of colorectal cancer (CRC) and triple-negative breast cancer(b (TNBC), EV-miR-15a and EV-miR-424-5p respectively, lead to a reduction in PD-L1 expression within recipient cells [89–91]. Considering the dual function of TEV-ncRNAs in controlling PD-1/PD-L1 expression, future studies in the field of tumor therapy should aim to exploit their immunostimulatory potential while avoiding the immunosuppressive impacts of EVs.

### Functions and mechanisms of the PD-1/PD-L1 pathway.

Immune checkpoint proteins (ICPs), including PD-1 and its cognate PD-L1, act as critical modulators of the immune system and are indispensable for preserving self-tolerance and regulating immune reactions [92]. PD-1, also designated cluster of differentiation 279 (CD279), is a 55 kDa type I transmembrane glycoprotein consisting of 288 amino acids. Its structural features include an N-terminal extracellular domain, a transmembrane region, and both N- and C-terminal intracellular tails [43]. The receptor PD1 is a member of the CD28 family [93]. PD-1 is expressed on the surface of immune cells, such as T cells, B cells, and myeloid cells, and serves as a biomarker for exhausted T cells [94]. As a ligand for PD-1, PD-L1 (also known as B7 homolog 1 or CD274) represents a significant barrier to antitumor immunity. Engagement of PD-L1 with PD-1 triggers the assembly of the PD-1/TCR (T cell receptor) inhibitory microcluster, which recruits SHP1/2 molecules. This interaction dephosphorylates several elements of the TCR signaling cascade, causing T cell dysfunction, exhaustion, anergy, or apoptosis, and ultimately facilitating cancer cell immune evasion [95]. The PD-1/PD-L1 signaling pathway is essential for preventing autoimmune responses by inhibiting the activation and proliferation of T-cells, which are crucial components of the immune system. However, this pathway also regulates antitumor immunity, which many cancer cells exploit to escape T-cell-mediated immune surveillance [96].

Tumor immune escape is a critical strategy for the survival and proliferation of tumor cells and involves multiple mechanisms. In recent years, the PD-L1/PD-1 pathway has been extensively studied as a central regulatory factor in tumor escape [97]. Consequently, the inhibition of the PD-L1/PD-1 pathway has surfaced as an attractive therapeutic approach in cancer treatment, presenting new opportunities to improve patient outcomes [98]. T cell exhaustion can be reversed by disrupting PD1/PD-L1 interactions [99, 100]. For instance, in clinical practice, monoclonal antibodies that target PD-1 (such as pembrolizumab and nivolumab) and PD-L1 (including atezolizumab and durvalumab) have shown considerable effectiveness across a range of malignancies, including melanoma, non-small cell lung cancer (NSCLC), and renal cell carcinoma (RCC) [101]. Recent research has found that the gut microbiota exerts a significant impact on the therapeutic efficacy of PD-1/PD-L1 inhibitors in solid tumors [102]. Additionally, under chronic stimulation or hypoxic conditions, CD8 + T cells develop mitochondrial dysfunction and accumulate large amounts of reactive oxygen species (ROS), leading to an exhaustion-like state [103]. Consequently, elevated ROS levels and the resulting oxidative stress regulate T-cell activity and resistance to anti-PD-L1 therapy [104]. Furthermore, V-domain immunoglobulin suppressor of T-cell activation (VISTA) or programmed death-1 homolog (PD-1H), a member of the CD28/B7 family, functions as an immune checkpoint and negative immune regulator. Combining anti-VISTA therapy with appropriate ICIs enhances the immunogenicity of tumors by amplifying T-cell effector activity, thereby achieving more pronounced therapeutic effects [105]. These ICIs revitalize exhausted T cells, thereby improving their capacity to identify and eliminate tumor cells [106, 107]. Nevertheless, the significant side effects associated with these synthetic antibodies still limit their clinical use in humans [108]. In contrast, the double lipid membrane structure of exosomes reduces immunogenicity and efficiently transfers molecules to target cells. These findings emphasize the potential of exosome-based therapeutics acting as checkpoint inhibitors to activate T cell immune responses, providing a viable strategy for successful immunotherapy. In the following chapters, the manner in which three distinct EV-ncRNAs species modulate the PD-1/PD-L1 pathway across various tumor types through unique molecular mechanisms, ultimately resulting in phenotypic outcomes such as tumor growth, immune evasion, or suppression of tumor advancement, will be elaborated upon. Additionally, the prospective clinical utility of EV-ncRNAs as biomarkers for cancer detection and as potential therapeutic targets in the realm of immunotherapy will be explored.

## EV-ncRNAs act as key players in the PD-1/PD-L1 pathway.

### EV-miRNAs modulate the PD-1/PD-L1 pathway

**Fig. 2:**
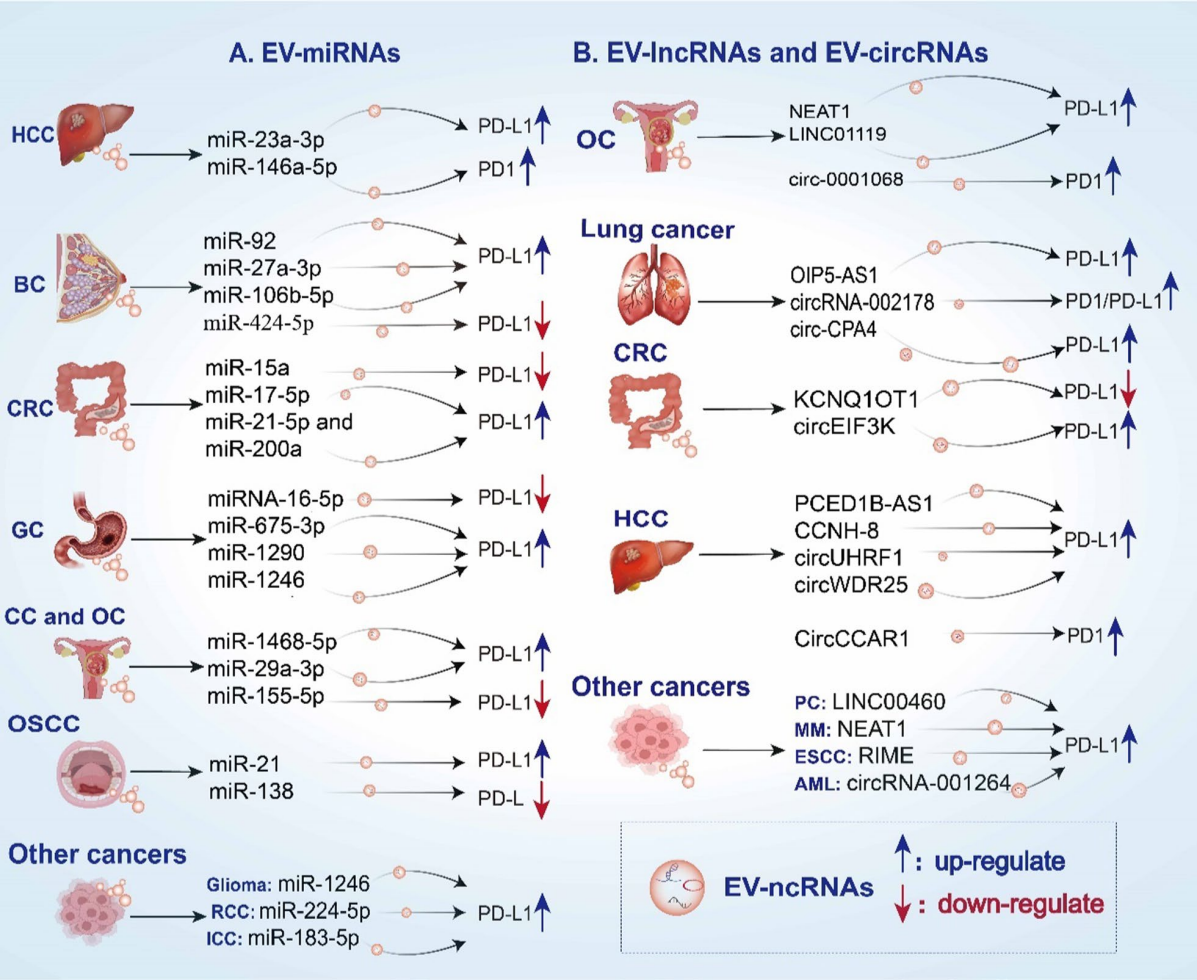
Regulatory relationships between different EV-ncRNAs and PD-1/PD-L1 expression in various cancer types. **A** The regulation of PD-1/PD-L1 expression by EV-miRNAs in GC, HCC, BC, melanoma, OC, NSCLC, and TNBC; **B** The regulation of PD-1/PD-L1 expression by EV-circRNAs and EV-lncRNAs in GC, HCC, BC, melanoma, OC, NSCLC, and TNBC

Numerous studies have shown that EV-miRNAs can influence the PD-1/PD-L1 pathway through both direct and indirect mechanisms in various cancers, such as CRC, glioma, GC (GC), acute myeloid leukemia (AML), and melanoma. In the following sections, we examine how EV-miRNAs regulate the PD-1/PD-L1 pathway in various tumors and explore the complex interactions between these molecules and the TME (Table 1 and Fig. 2A).

**Table 1:** Summary of EV-ncRNAs regulating the PD-1/PD-L1 pathway in cancer

EV-ncRNAs	Expression	Source cell	Receptor cells	Target/Pathway	PD-1/PD-L1 Expression	Functions	References
EV-miRNAs in PD-1/PD-L1 Pathways							
MiR-23a-3p	↑	HCC	macrophage	PTEN/AKT	PD-L1↑	Suppressed PTEN expression accompanied by increased phosphorylation of AKT and elevated PD-L1 levels	[113]
MiR-146a-5p	↑	HCC	T cell	SALL4/miR-146a-5p/PD-1	PD-1↑	Expression of PD-1 and CTLA-4 was elevated in T cells	[114]
MiR-1468-5p	↑	Cervical cancer	LECs	iR-1468-5p/SOCS1/HMBOX1/JAK2/STAT3	PD-L1↑	Enhances lymphatic PD-L1 expression and induces lymphangiogenesis, leading to the suppression of T cell immunity	[125]
MiR-29a-3p	↑	TAM	OC	MiR-29a-3p/FOXO3-AKT/GSK3β/PD-L1	PD-L1↑	Elevates PD-L1 expression to promote OC cell proliferation and enable immune evasion	[126]
MiR-155-5p	↓	OC	Macrophages	Exo-miR-155-5p/PD-L1	PD-L1↓	Exo-miR-155 administration reduces PD-L1 expression in macrophages	[127]
MiR-21	↑	OSCC	MDSCs	MiR-21/PTEN/PD-L1	PD-L1↑	MiR-21 upregulates PD-L1 expression in MDSCs through the PTEN signaling pathway	[134]
MiR-138	↑	γδ T cell	CD8 + T cells	MiR-138/PD-1	PD-1↓	Decreased the expression of PD-1 and CTLA-4 and increased cytotoxicity of CD8 + T cells against OSCC cells	[133]
MiR-424-5p	↑	AT-MSCs	TNBC	MiR-424-5p/PD-L1	PD-L1↓	EVs-miR-424 suppresses PD-L1 expression in target cells	[122]
MiR-92	↑	CAF	BC	MiR-92/LATS2/YAP1/PD-L1	PD-L1↑	Enhanced PD-L1 expression promotes T cell apoptosis and impairs their proliferation	[121]
MiR-27a-3p	↑	BC	Macrophages	MiR-27a-3p/MAGI2/PTEN/PI3K	PD-L1↑	Facilitates immune evasion by modulating PD-L1 expression in macrophages	[118]
MiR-106b-5p /MiR-18a-5p	↑	BC	TAMs	MiR-106b-5p/PTEN/AKT/PD-L1/miR-18a-5p/PIAS3/STAT3/PD-L1	PD-L1↑	Synergistically enhanced PD-L1 expression in M2 TAMs, leading to increased invasion and metastasis of BC cells	[120]
MiR-15a	↑	adMSCs	CRC	KDM4B/HOXC4/PD-L1	PD-L1↓	Inhibited immune escape in CRC by modulating the KDM4B/HOXC4/PD-L1 pathway	[89]
MiR-17-5p	↑	CRC	CRC	MiR-17-5p/SPOP/PD-L1	PD-L1↑	MiR-17-5p directly targets SPOP, resulting in PD-L1 upregulation, thereby modulating anti-tumor immunity in CRC cells	[132]
MiR-21-5p and MiR-200a	↑	CRC	Macrophage	PTEN/AKT and SCOS1/STAT1	PD-L1↑	Induces M2-like macrophage polarization and PD-L1 upregulation, reducing CD8 + T cell function and promoting tumor growth	[131]
MiR-16-5p	↑	M1 Macrophages	GC	MiRNA-16-5p/PD-L1	PD-L1↓	Suppressed anti-tumor immune response and tumor development in vivo through the downregulation of PD-L1 expression	[112]
MiR-675-3p	↑	GC	GC	MiR-675-3p/CXXC4/MAPK/PD-L1	PD-L1↑	Upregulates PD-L1 expression, promoting immune escape in GC	[110]
MiR-1290	↑	GC	GC	MicroRNA-1290/Grhl2/ZEB1/PD-L1	PD-L1↑	Upregulates PD-L1 to potentiate the inhibitory function of GC cells on T cell activation	[109]
MiR-1246	↑	GC	LEC	MiR-1246/GSK3β/β-Catenin/MMP7 /PD-L1	PD-L1↑	Promotes lymphangiogenesis and enhances PD-L1 expression in LECs	[111]
MiR-1246	↑	Glioma	MDSC	POU5F1/miR-1246 /DUSP3/ERK /M-MDSCs/PD-L1	PD-L1↑	MiR-1246 orchestrates the differentiation and functional activation of MDSCs	[136]
MiR-224-5p	↑	RCC	RCC	miR-224-5p/CCND1/PD-L1	PD-L1↑	MiR-224-5p augments the stability of PD-L1 protein through the suppression of CCND1	[137]
MiR-183-5p	↑	ICC	Macrophage	MiR-183-5p/PTEN/AKT/PD-L1	PD-L1↑	Upregulates PD-L1-expressing macrophages, fostering immunosuppression and accelerating the progression of ICC	[138]
EV-LncRNAs in PD-1/PD-L1 Pathways							
NEAT1	↑	Macrophages	OC	NEAT1/miR-101-3p/ZEB1/PD-L1	PD-L1↑	NEAT1 sponged miR-101-3p to enhances ZEB1 and PD-L1 expression	[150]
LINC01119	↑	Adipocytes	OC	LINC01119/ SOCS5	PD-L1↑	Upregulating PD-L1, and reducing T cell cytotoxicity against SKOV3 cells	[149]
NEAT1	↑	Multiple myeloma	multiple myeloma	NEAT1/EZH2/PBX1/PD-L1	PD-L1↑	Modulates the EZH2/PBX1 axis to suppress NK-cell activity	[151]
LINC02096 (RIME)	↑	ESCC	ESCC	RIME-MLL1-H3K4me3/PD-L1	PD-L1↑	Elevates PD-L1 and IDO-1 expression in tumor cells while suppressing CD8 T cell infiltration and activation	[154]
LINC00460	↑	PC	PC	LINC00460/miR-503-5p/ANLN	PD-L1↑	Silencing LINC00460 diminishes tumor growth in PC via enhanced response to anti-PD-1 therapy	[152]
LncRNA KCNQ1OT1	↑	CRC	CRC	LncRNA KCNQ1OT1/MiR-30a-5p/USP22/PD-L1	PD-L1↓	Regulates PD-L1 ubiquitination through miR-30a-5p/USP22 to promote colorectal cancer immune escape	[153]
PCED1B-AS1	↑	HCC	HCC	PCED1B-AS1/hsa-miR-194-5p/PD-1	PD-L1↑	Enhances the expression and function of PD-Ls to induce immunosuppression in HCC	[148]
Lnc-CCNH-8	↑	HCC	HCC	Lnc-CCNH-8/miR-3173/PKP3/PD-L1	PD-L1↑	Inducing immune escape from CD8 + T-cell-mediated killing by up-regulating PD-L1 in a miR-3173-dependent manner	[147]
OIP5-AS1	↑	CAFs	Lung cancer	OIP5-AS1/miR-142-5p/ PD-L1	PD-L1↑	promoted tumor growth through decreasing miR-142-5p and up-regulating PD-L1	[155]
EV-circRNAs in PD-1/PD-L1 Pathways							
CircRNA-002178	↑	LUAD	Cancer cells/CD8 + T	CircRNA-002178/miR-34/PD-L1/PD-1	PD-1↑/PD-L1↑	CircRNA-002178 could act as a ceRNA to promote PD-L1/PD-1 expression	[161]
CircCCAR1	↑	HCC	CD8 + T cells	CircCCAR1/miR-127-5p/WTAP	PD-1↑	Caused dysfunction of CD8 + T cells by stabilizing the PD-1 protein	[158]
CircUHRF1	↑	HCC	HCC	CircUHRF1/miR-449c-5p/TIM-3/anti-PD-1	PD-L1↑	Forced expression of circUHRF1 potentially obstructs HCC response to anti-PD-1 treatment	[181]
CircWDR25	↑	HSC	HCC	CircWDR25/miR-4474-3p/ALOX15/PD-L1	PD-L1↑	Enhanced expression of CTLA-4 in HSCs and PD-L1 in HCC cells was observed	[160]
CircRNA-001264	↑	AML	M2 macrophage	CircRNA-001264 /p38-STAT3/PD-L1	PD-L1 ↑	Facilitates immunosuppression in AML by promoting M2-like macrophage polarization and PD-L1 upregulation	[163]
CircEIF3K	↑	CAF	CRC	CircEIF3K/miR-214/PD-L1	PD-L1↑	Promotes CRC progression via miR-214/PD-L1 axis	[164]
Circ-0001068	↑	OC	T cells	Circ-0001068/miR-28-5p /PD-1	PD-1↑	Upregulation expression of PD-1 and T cell exhaustion	[162]

#### Gastric cancer (GC)

GC is the fifth most common malignancy globally, accounting for 5.7% of all cases among men and women. Unfortunately, despite advancements in medical treatments, GC remains a major cause of cancer death, accounting for approximately 8.2% of total fatalities [109]. Currently, numerous studies have shown that different miRNAs contained in EVs released by GC cells are involved in immune evasion associated with GC. MiR-16-5p, miR-675-3p, miR-1290, and miR-1246 have been identified as key players in immune evasion, specifically by modulating the PD1/PD-L1 pathway [110]. For example, high levels of miR-1246 are found in the plasma EVs of *H. pylori*-positive GC patients, indicating a poor prognosis. Functionally, EVs-miR-1246 stimulate lymphangiogenesis and lymphatic remodeling. Mechanistically, miR-1246 downregulates GSK3β and promotes β-catenin and downstream MMP7 expression in lymphatic endothelial cells (LECs). Notably, miR-1246 also stabilizes PD-L1 and induces apoptosis in CD8 + T cells via GSK3β inhibition [111]. Moreover, Liang et al. reported that EV-miRNA-1290 promotes immune evasion in GC cells through the modulation of the Grhl2/ZEB1/PD-L1 axis. Notably, the critical function of EV-miR-1290 as an oncomiR was validated in vivo [109]. EV-miR-675-3p inhibits the expression of the target gene CXXC4 and enhances PD-L1 expression via the MAPK signaling pathway, ultimately stimulating immune escape in GC cells [110]. Additionally, certain exosomal miRNAs have been identified as significant inhibitors of GC progression. MiR-16-5p, which is found in exosomes from M1 macrophages, plays a crucial role in slowing GC development. This occurs by reducing PD-L1 expression and enhancing T-cell-mediated immune responses [112].

These results highlight the potential of EV-miRNAs as effective targets in PD-1/PD-L1 checkpoint blockade therapy for GC, while the transfer of miRNAs via EVs to target genes and PD-L1 also shows promise as a diagnostic tool for this disease. However, creating clinical protocols for EV-based diagnosis, prognosis, and treatment necessitates a deeper understanding of EVs' roles and mechanisms in GC. This understanding is crucial for advancing these approaches.

#### Hepatocellular carcinoma (HCC)

Recent studies have revealed a strong positive link between certain EV-miRNAs, including miR-23a-3p and miR-146a-5p, and PD-1/PD-L1 activity in HCC. Notably, Exosomes from HCC cells experiencing endoplasmic reticulum (ER) stress contain elevated levels of miR-23a-3p. When these exosomes were introduced, PD-L1 expression increased in macrophages. Further research revealed that miR-23a-3p regulates PD-L1 expression via the PTEN-PI3K-AKT signaling pathway. As a result, this regulation led to the suppression of T-cell function [113]. Furthermore, macrophages influenced by miR-146a-5p from HCC-related exosomes reduced the levels of IFN-γ and TNF-α, while increasing the expression of inhibitory receptors like PD-1 and CTLA-4 in T cells. In mechanism, the transcription factor Sal-like protein-4 (SALL4) binds to the promoter of miR-146a-5p, directly regulating its expression in exosomes. Disrupting the SALL4/miR-146a-5p interaction in HCC led to decreased expression of inhibitory receptors on T cells, This effectively reversed T-cell exhaustion and slowed the progression of HCC in DEN/CCL(4)-induced murine models [114].

These investigations offer valuable perspectives on how tumor cells evade antitumor immune responses, indicating possible therapeutic and diagnostic targets for HCC. Furthermore, these findings highlight a new target for the prevention and treatment of HCC.

#### Breast cancer (BC)

TNBC accounts for approximately 15–20% of all BC cases. It is characterized by aggressive behavior, poor prognosis, high recurrence rates, and a tendency to metastasize, especially to the lungs and brain [115, 116]. Recent studies have shown that several miRNAs from EVs, such as EV-miR-27a-3p, EV-miR-106b-5p, and EV-miR-18a-5p, significantly upregulate PD-L1 expression in tumor-associated macrophages (TAMs). These miRNAs not only enhance tumor cell proliferation in vitro but also promote tumor growth, invasion, and metastasis in vivo [117]. For example, exosomal miR-27a-3p, which is induced by ER stress, promotes immune evasion in BC by modulating PD-L1 expression in macrophages. This miRNA affects MAGI2, which triggers the PTEN-AKT/PI3K pathway. As a result, PD-L1 levels rise on macrophages, causing a decrease in the number and effectiveness of CD4 + and CD8 + T cells [118]. The investigation revealed a connection between elevated TAM levels and a reduced M1/M2 TAM ratio in BC. This finding indicates a poor prognosis [119]. A clinical study reveals that that EV-miR-106b-5p and EV-miR-18a-5p were related to the development of BC metastasis and poor prognosis in BC patients. Furthermore, mechanistic experiments revealed that BC-derived EV-miR-106b-5p and EV-miR-18a-5p synergistically upregulated PD-L1 expression in M2 TAMs by modulating the PTEN/AKT and PIAS3/STAT3 pathways, leading to enhanced BC cell invasion and metastasis[120]. Moreover, BC-derived fibroblasts associated with cancer (CAFs) secrete exosomal miR-92, leading to increased PD-L1 expression in BC cells. Consequently, it hinders T-cell proliferation and facilitates immune evasion [121]. In contrast, specific miRNAs transported via EVs have been recognized as significant inhibitors of PD-L1 signaling in BC. For instance, EV-miR-424-5p has been demonstrated to inhibit PD-L1 signaling, promote an inflammatory microenvironment in TNBC, increase the apoptosis of tumor cells in vitro, and restrain tumor progression in vivo. These observations indicate that EVs might function as an effective delivery mechanism for innovative immunotherapies aimed at the miR-424-5p/PD-L1 pathway in TNBC [122].

Together, by identifying several mechanisms through which miRNAs promote immune evasion in breast cancer, these studies highlight the potential treatment strategy of targeting EV-miRNAs and the PD-L1 pathway.

#### Gynecological cancer (GC)

Most early-stage cervical cancer (CCa) patients respond well to surgery and concurrent chemoradiotherapy. However, effective treatments are limited for those with recurrent or metastatic cervical cancer [123]. Recent reports emphasize immune checkpoint blockade as a promising approach to enhance T cell function and improve treatment efficacy, especially in patients with recurrent or metastatic cervical cancer, where effective treatments are scarce [124].

In cervical cancer, different EV-ncRNAs play crucial roles in promoting cancer progression and facilitating immunosuppression induced by PD-1/PD-L1. For instance, exosomes containing miR-1468-5p increase the expression of PD-L1 in lymphatic endothelial cells. This process promotes lymphangiogenesis and weakens T cell-mediated immune responses. Specifically, it activates the JAK2/STAT3 signaling pathway in lymphatic endothelial cells by targeting HMBOX1 in the promoter region of SOCS1, which contributes to the formation of an immunosuppressive microenvironment. This process not only stimulates lymphangiogenesis but also reduces T cell-mediated immune responses, creating a more favorable environment for immune evasion [125]. Similarly, exosomal miR-29a-3p from TAMs boosts PD-L1 expression, promoting the growth of ovarian cancer (OC) cells and their ability to escape immune responses. Notably, inhibiting exosomal miR-29a-3p reduces PD-L1 expression, thereby hindering OC progression via the FOXO3-AKT/GSK3β signaling pathway [126]. Additionally, unlike the previously mentioned miRNAs, EVs can influence the strong tumor-suppressive immune responses of CD8 + T cells by delivering specific miRNAs. For example, exosomal miR-155-5p targets PD-L1. This action increases CD8 + T lymphocyte levels and decreases T cell apoptosis. Exosomes derived from NAC were shown to inhibit tumor growth in nude mice and suppress OC in immunocompetent mice. Notably, exosomal miR-155-5p exhibited a stronger inhibitory effect on tumor growth than PD-L1 antibodies did [127].

Gaining insights into how these miRNAs function and are regulated may pave the way for new treatments that can more effectively inhibit the progression and metastasis of CCa.

#### Colorectal cancer (CRC)

CRC is one of the most common cancers worldwide [128]. CRC develops and progresses through genetic and epigenetic mechanisms, and it is also closely linked to the TME, especially the tumor immune microenvironment (TIME) [129]. ICB therapy is approved for treating CRC patients with DNA mismatch repair deficiency (dMMR) and high microsatellite instability (MSI-H). However, this subgroup accounts for only 15% of CRC patients, leaving the majority unresponsive to ICB treatment [130]. Therefore, understanding the dynamic interactions between CRC and the immune system, as well as developing strategies to modulate immune cells in the TME, could lead to promising new pathways for CRC immunotherapy.

CRC-derived EVs rich in miR-21-5p and miR-200a work together to promote M2-like TAM polarization and increase PD-L1 expression through the PTEN/AKT and SOCS1/STAT1 signaling pathways. This modulation enhances TAM-mediated suppression of CD8 + T cells, thereby promoting immune evasion and accelerating CRC progression [131]. Another study revealed that exosomal miR-17-5p derived from tumor stem cells impairs tumor-suppressive immunity in CRC by interfering with SPOP and enhancing PD-L1 expression [132]. Conversely, some exosomal miRNAs act as important inhibitors that promote apoptosis in CRC cells and help prevent immune escape. For example, EVs from adipose mesenchymal stem cells (AdMSCs) that overexpress miR-15a reduce CRC immune evasion by affecting the KDM4B/HOXC4/PD-L1 pathway [89].Thus, reducing EV-miR-21-5p and EV-miR-200a or increasing EV-miR-15a could be a promising methods for immunotherapy in CRC patients.

#### Oral squamous cell carcinoma (OSCC)

In OSCC, two specific exosomal miRNAs from γδ T cells and hypoxic OSCC cells play a vital roles in regulating the PD-1/PD-L1 pathway. In particular, EVs derived from γδ T cells (γδTDEs) transfer miR-138 to OSCC cells, leading to significant downregulation of PD-1 and CTLA-4 expression at both the transcriptional and protein levels. This downregulation strongly inhibits the growth and survival of OSCC cells, as shown in cell culture and animal studies [133]. In contrast, exosomal miR-21 from hypoxic OSCC cells increases PD-L1 expression in myeloid-derived suppressor cells (MDSCs) via the PTEN signaling pathway. The increased PD-L1 on MDSCs interacts with PD-1 on γδ T cells. This interaction suppresses the immune function of γδ T cells [134].

These findings highlight the complex roles of EV-miRNAs in the OSCC tumor microenvironment, showing their ability to both inhibit and promote tumor growth by modulating the PD-1/PD-L1 axis. Therefore, understanding this duality is crucial for developing effective miRNA-based therapies that target immune checkpoints in cancer treatment.

#### Other cancers

Exosomal miR-1246 from glioma patients enhances the activation of MDSCs. These MDSCs express high levels of PD-L1, leading to T cell exhaustion [135, 136]. Furthermore, miR-224-5p, produced by RCC cells, ncreases PD-L1 expression by inhibiting cyclin D1. Consequently, this finding indicates that miR-224-5p holds promise as a biomarker for predicting the effectiveness of PD-1/PD-L1 blockade therapies [137]. Furthermore, exosomal miR-183-5p from intrahepatic cholangiocarcinoma (ICC) cells decreases PTEN levels in macrophages. This reduction leads to increased PD-L1 expression and impaired T cell immunity, which is associated with a poor prognosis in ICC patients [138]. Exosomal miR-16-5p may suppress tumor growth and could be a potential biomarker for immunotherapy using PD-L1 inhibitors in lung adenocarcinoma [139]. In conclusion, these results indicate that the interaction between EV-miRNAs and target genes can regulate the PD-1/PD-L1 pathway, potentially contributing to cancer development and immune evasion (Fig. 2A and Table 1). 

### EV-lncRNAs modulate the PD-1/PD-L1 pathway

Most lncRNAs are found in the nucleus, where they play roles in transcription regulation and mRNA degradation [140, 141]. Cytoplasmic lncRNAs chiefly engage in posttranscriptional regulation, operating as miRNA sponges to repress miRNAs or as competitive endogenous RNAs (ceRNAs) to regulate gene expression posttranscriptionally [142–144]. In addition to EV-miRNAs, EVs orchestrate immune responses in different tumors by transferring a series of lncRNAs, including the modulation of the PD-1/PD-L1 axis [145, 146]. These EV-lncRNAs are summarized in Table 1 and Fig. 2B.

For example, in HCC, exosomal Lnc-CCNH-8 and PCED1B-AS1 orchestrate different T cell subsets, thereby impairing antitumor immunity. Functional analyses revealed that elevated Lnc-CCNH-8 levels impair T cell activity in vitro and compromise antitumor immunity in immunocompetent mice. Mechanistically, Lnc-CCNH-8 stabilizes PD-L1 via the miR-3173/PKP3 pathway. Strikingly, tumors with high exosomal Lnc-CCNH-8 expression exhibit increased sensitivity to anti-PD-L1 monoclonal antibody therapy, underscoring its potential as a predictive immunotherapy response biomarker in HCC [147]. Furthermore, EV-lncRNAs can be transmitted to neighboring immune-related cells, thereby concurrently inhibiting the function of recipient T lymphocytes and macrophages and inducing a phenotypic shift toward tumor immunosuppression. For example, exosomal PCED1B-AS1 enhances PD-L1 expression and activity by sequestering miR-194-5p, concurrently inhibiting the function of recipient T lymphocytes and macrophages. Notably, PCED1B-AS1 also promotes cell proliferation and colony formation both in vivo and in vitro [148]. Zheng *et al*. reported that the level of cancer-associated adipocyte (CAA)-derived exosomal LINC01119 is increased in OC patients and that this elevation is associated with shorter overall survival. Mechanistically, exosomal LINC01119 facilitates immune evasion in OC by promoting M2 macrophage polarization via SOCS5 upregulation. This is evidenced by reduced CD3+ T cell proliferation, elevated PD-L1 expression, and diminished T cell cytotoxicity against SKOV3 cells [149]. Exosomal NEAT1 has been established as an oncogene in OC and multiple myeloma. Yin *et al*. reported that NEAT1 was highly expressed in M2-derived EVs and in OC cells cocultured with these EVs. Mechanistically, NEAT1 sequestered miR-101-3p, increasing ZEB1 and PD-L1 expression. Both in vitro and in vivo experiments corroborated the tumor-promoting activities of EV-NEAT1, which increased OC cell proliferation, induced CD8+ T-cell apoptosis, and facilitated tumor growth [150]. Exosomal NEAT1 from myeloma cells modulates EZH2/PBX1, impairing NK-cell function and promoting immune evasion. NEAT1-silenced exosomes inhibit tumor growth, reduce Ki67 and PD-L1 expression, and increase NKG2D, TNFα, and IFNγ expression in tumors [151]. Additionally, an independent study demonstrated increased levels of exosomal LINC00460 in PC cells and tissues, acting as an endogenous sponge for miR-503-5p and targeting ANLN. Notably, depletion of LINC00460 impeded PC tumor progression through anti-PD1 immunotherapy and increased the vulnerability of PANC-1 cells to CD8+ T cell-mediated cytotoxicity [152]. The lncRNA KCNQ1OT1, which is secreted by exosomes derived from tumor cells, facilitates immune escape in colorectal cancer by regulating PD-L1 ubiquitination through the miR-30a-5p/USP22 pathway [153].

In patients with ESCC, increased expression of the lncRNA LINC02096, also known as RIME, in plasma-derived exosomes is associated with a diminished response to PD-1 monoclonal antibody (mAb) therapy and an adverse prognosis. Mechanistically, RIME stabilizes MLL1 by inhibiting ubiquitination mediated by ASB2, leading to an increase in H3K4me3 levels at the promoters of PD-L1 and PD1. This, in turn, upregulates their expression and suppresses the infiltration of CD8(+) T cells. Strikingly, the depletion of the RIME in huPBMC-NOG mice retarded tumor growth and augmented the effectiveness of the PD-1 mAb through T-cell activation. As such, the RIME is a promising prognostic biomarker for immunotherapy [154]. Exosomal OIP5-AS1 in lung cancer increases PD-L1 levels, inhibits T-cell proliferation, and attenuates T-cell toxicity in tumor cells, providing potential diagnostic and therapeutic opportunities [149–155].

Collectively, the aforementioned studies elucidated the extensive and pivotal contributions of EV-lncRNAs in fostering tumor cell proliferation, immune cell apoptosis, and overall tumor growth through the induction of PD-L1 expression. Consequently, unraveling the underlying mechanism behind the increase in PD-L1 expression mediated by exosomal lncRNAs will expedite the identification and advancement of innovative ICIs that target these lncRNAs, revealing a promising therapeutic approach for the management of such neoplasms.

### EV-circRNAs modulate the PD-1/PD-L1 pathway

CircRNAs function as miRNA sponges, thereby alleviating the inhibitory effects of miRNAs on their target genes [156, 157] Numerous circular RNAs act as ceRNAs, regulating PD-L1 abundance and thereby mediating tumor immune avoidance in various cancers. Moreover, numerous EV-circRNAs increase the levels of immune checkpoint proteins, including PD-1/PD-L1, in both tumor and immune cells via diverse cytokine-mediated signaling pathways, thereby modulating the capacity of immune cells to combat tumors. How do exosomal circRNAs regulate the PD-1/PD-L1 pathway? The following sections provide a detailed description (Table 1 and Fig. 2B).

Multiple studies have demonstrated that EV-circRNA dysregulation is involved in the proliferation, invasion, and immune evasion of HCC. For example, elevated circCCAR1 levels were detected in HCC tissues, plasma exosomes, culture supernatants, and cells. CircCCAR1 stimulated on the growth and metastasis of HCC both in vitro and in vivo. Mechanistically, the stability of circCCAR1 was augmented through m6A modification mediated by Wilms tumor 1-associated protein (WTAP), which achieved this by binding to insulin-like growth factor 2 mRNA-binding protein 3 (IGF2BP3). CircCCAR1 functions as a molecular sponge for miR-127-5p, thereby upregulating its target WTAP, leading to the formation of a feedback loop consisting of the circCCAR1/miR-127-5p/WTAP axis. Additionally, exosomal circCCAR1 was internalized by CD8 + T cells, resulting in their dysfunction by stabilizing the PD-1 protein [158]. Circ_0032704 was notably overexpressed in sorafenib-resistant HCC tissues and cell lines. Additionally, exosomal delivery of circ_0032704 was shown to confer resistance to sorafenib in HCC and promote its malignant progression via modulation of the miR-514a-3p/PD-L1 signaling pathway [159]. Furthermore, exosome-derived circWDR25 from HSCs facilitates HCC cell proliferation and invasion through the circWDR25/miR-4474-3p/ALOX15 axis, concurrently increasing CTLA-4 expression in HSCs and PD-L1 levels in HCC cells [160].

In Lung Adenocarcinoma (LUAD,) exosomal circRNA-002178 enhances PD-1 expression through the sequestration of miR-28-5p in CD8 + T cells. Notably, circRNA-002178 was detectable within the plasma-derived exosomes of LUAD patients, indicating its potential as a biomarker for the early identification of this disease [161]. Wang et al. reported significant upregulation of circ-0001068 in the serum-derived exosomes of OC patients. This exosome-encapsulated circ-0001068 is transferred to T cells, where it sponges miR-28-5p, resulting in PD-1 upregulation and T-cell exhaustion. These observations suggest that circ-0001068 is a potential noninvasive biomarker and therapeutic candidate for OC diagnosis and therapy [162]. Exosomal circ_001264 enhances crosstalk between macrophages and AML cells and is associated with poor prognosis. Du et al. reported that exosomal circ_001264 from AML cells regulates RAF1, activates p38-STAT3, and polarizes M2 macrophages to overexpress PD-L1. In mouse models, combined siRNA and anti-PD-L1 treatment reduced the leukemia burden [163]. Hypoxia, a prominent hallmark of the TME, substantially influences cancer aggressiveness and the therapeutic response. Under hypoxia, cancer-associated fibroblasts secrete exosomes enriched with circEIF3K. These exosomes promote CRC progression through the miR-214/PD-L1 pathway [164].

These results indicate that EV-circRNAs play an indispensable role in modulating immune checkpoint expression [165]. (Table 1 and Fig. 2B) Given their abundance and role in intercellular communication, EV-circRNAs directly or indirectly remodel the TME, significantly impacting both immune cells and immune checkpoint molecules.

## Potential clinical applications of EV-ncRNAs in anti-PD-1 /PD-l1 cancer immunotherapy.

The advent of ICIs, particularly anti-PD-1/PD-L1 therapies, has revolutionized cancer treatment by enhancing antitumor immunity. Despite their success in treating various cancers, these therapies are only beneficial for a limited number of patients due to both inherent and acquired resistance mechanisms. As mentioned earlier, extracellular vesicle non-coding RNAs (EV-ncRNAs) play a vital role in regulating the PD-1/PD-L1 axis, impacting tumor immune evasion and treatment effectiveness. EV-ncRNAs hold promise as novel biomarkers and therapeutic targets, which could optimize anti-PD-1/PD-L1 therapy and extend its clinical application [166].

### EV-ncRNAs serve as promising biomarkers

**Figure3:**
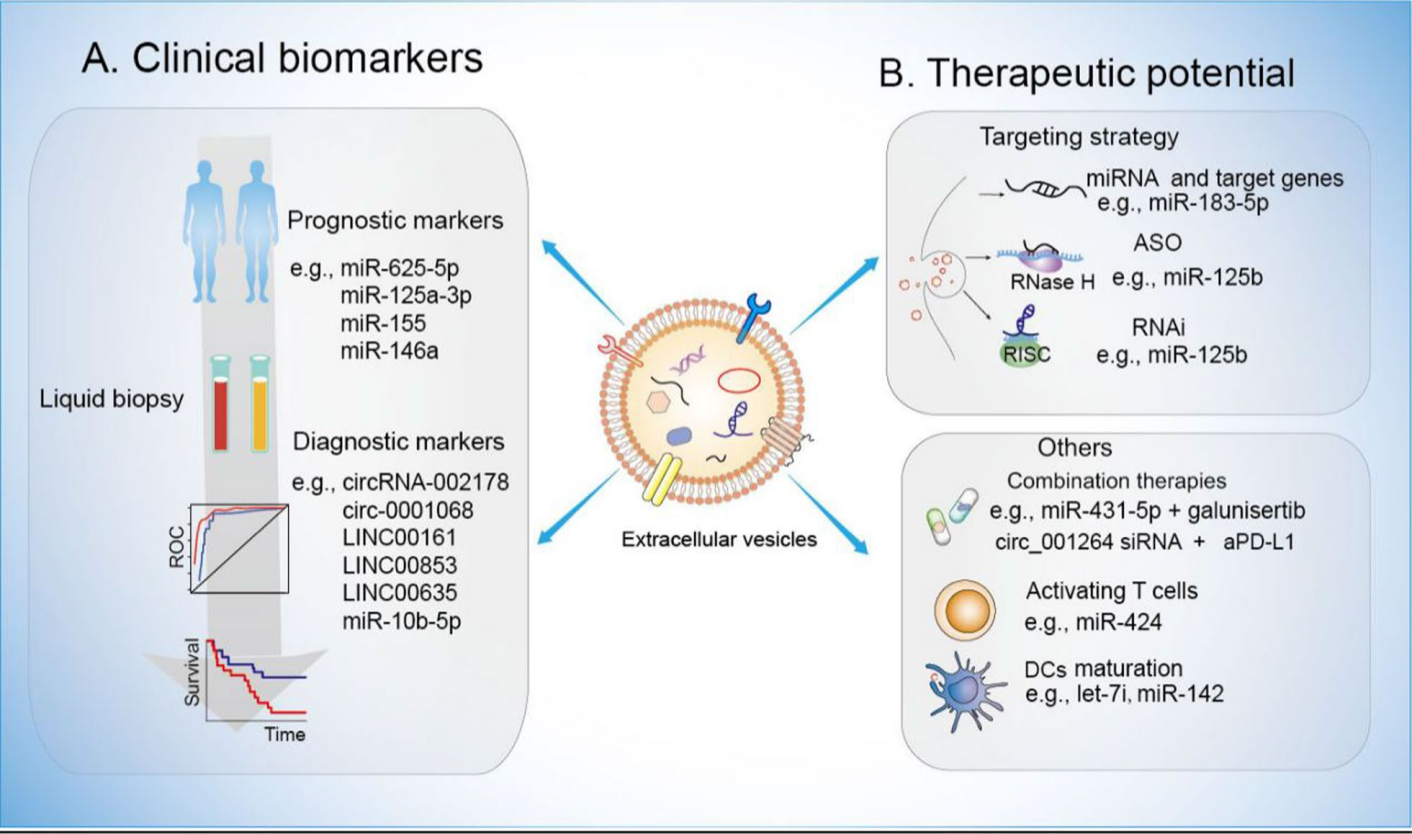
Potential clinical applications of EV-ncRNAs in cancer immunotherapy. **A** EV-ncRNAs serve as promising biomarkers: Diagnosis and prognosis. These EV-ncRNAs can be measured from liquid biopsy samples and are novel noninvasive diagnostic and prognostic biomarkers. **B** Targeting strategies. Knockdown technologies can be used to target ncRNAs. Combination therapies. Combining therapies targeting EV-ncRNAs with anti-PD-1/PD-L1 immunotherapy. RNAi, RNA interference; ASO, antisense oligonucleotides; RISC, RNase H, Ribonuclease H

EVs exhibit natural stability in biological fluids, safeguarding their cargo from degradation, and can be easily isolated and analyzed, rendering them ideal markers for cancer diagnosis and prognosis. Recent studies have demonstrated that the expression levels of EV-ncRNAs correlate with immunotherapy response in cancer patients, highlighting their potential as biomarkers for predicting response to immunotherapy (Fig. 3A). For instance, next-generation sequencing of plasma EV-miRNAs from advanced NSCLC patients treated with or without PD-1/PD-L1 inhibitors revealed a significant correlation between EV-miRNAs and anti-PD-1/PD-L1 treatment response [167–169]. Additionally, another clinical study on stage IV melanoma patients treated with nivolumab or ipilimumab (anti-PD-1) reported the overexpression of various circulating exogenous EV-miRNAs, such as miR-155, miR-146a, miR-125b, let-7e, miR-100, miR-125a, miR-99b, and miR-146b, which may predict responses to immunotherapy. Elevated levels of these miRNAs are associated with poor responses to anti-PD-1 drugs and shorter overall survival [170]. Pantano et al. investigated the levels of 799 EV-miRNAs in pretreatment plasma from 88 patients with advanced NSCLC receiving anti-PD-1 monotherapy. EV-miR-625-5p has emerged as an independent biomarker for response and survival in NSCLC patients receiving ICI treatment, particularly those with PD-L1 expression ≥ 50% [171]. Conversely, serum-derived exosomal miR-125a-3p serves as a potential predictor of the response to anti-PD-1/PD-L1 therapy in advanced NSCLC patients with low PD-L1 expression [172].

In addition to the prediction of responses to cancer immunotherapy emphasized in the aforementioned studies, the use of EV-ncRNAs as biomarkers for cancer diagnosis represents another crucial clinical application. As previously noted, the utilization of exosomes as delivery vectors for circRNA expression revealed that exosomal circRNA-002178 and circ-0001068 each hold potential as noninvasive biomarkers for the identification of LUAD and OC, respectively, and may also serve as immunotherapy targets [161, 162]. The area under the curve (AUC) for circRNA-002178 was 0.9956 (P < 0.001). Moreover, this study revealed that the level of circRNA-002178 in exosomes derived from cancer cells was significantly greater than that in exosomes from normal human bronchial epithelial cells. These findings further confirmed that serum exosomal circRNA-002178 can serve as a novel diagnostic biomarker for LUAD. Various EV-derived lncRNAs, including LINC00161, RNA ENSG00000258332.1, LINC00635, and miR-10b-5p, have recently been reported as diagnostic biomarkers for HCC [173–175]. The validated exosomal LINC00161 exhibited an area under the receiver operating characteristic (ROC) curve of 0.794 (95% CI 0.712–0.877) [174] whereas serum exo-miR-215-5p served as a prognostic biomarker for HCC [175]. Surprisingly, compared with the traditional biomarker AFP, EV-LINC00853 in HCC plasma exhibited better diagnostic value, especially for AFP-negative HCC, with an AUC of 0.934 (95% CI = 0.887–0.966) for EV-LINC00853 [176, 177].

Therefore, further research is urgently needed to identify key exosomal EV-ncRNA biomarkers that can predict early clinical responses to immunotherapy and assess the efficacy and safety of anti-PD-1/PD-L1 therapies. This will bridge the gap between basic research and clinical applications.

### EV-ncRNAs: key drivers of anti-PD-1 therapy resistance in cancer

Although PD-1/PD-L1 ICIs are highly effective in treating different cancers, their use is limited due to the development of drug resistance. In recent years, the role of EV-ncRNAs in cancer immunotherapy resistance has garnered considerable attention. These molecules inhibit the activation and function of immune cells, including CD8 + T cells and NK cells, through various mechanisms, creating an immunosuppressive environment that allows tumors to evade immune attacks. The following section expands on these molecular mechanisms and discusses cutting-edge strategies to disrupt EV-ncRNA-driven resistance.

For example, EV-circRNA can be taken up by CD8 + T cells, leading to CD8 + T cell dysfunction by stabilizing the PD-1 protein, thus promoting immune evasion and resistance to anti-PD-1 immunotherapy in HCC [158]. Hu et al. reported that high levels of exosomal circTMEM181 were found to promote an immunosuppressive microenvironment and confer anti-PD1 resistance in HCC. Mechanistically, exosomal circTMEM181 sponged miR-488-3p and upregulated CD39 expression in macrophages. Utilizing macrophage-specific CD39 knockout mice and pharmacological approaches, the study revealed a novel mode of anti-PD1 resistance in HCC. Specifically, the expression of CD39 in macrophages and CD73 in HCC cells worked together to activate the eATP-adenosine pathway. This activation led to increased adenosine production and impaired CD8 + T cell function, which contributed to anti-PD1 resistance [178]. Elevated levels of circUSP7 in lung cancer tissues are associated with poor clinical outcomes and CD8 + T cell dysfunction, which in turn promotes resistance to anti-PD-1 therapy. Notably, exosomal circUSP7 can be detected in peripheral blood, suggesting its potential as a noninvasive biomarker for predicting resistance to anti-PD-1 therapy in NSCLC patients [179]. In TNBC, exosomal miR-20a-5p is internalized by CD8 + T cells, resulting in CD8 + T-cell dysfunction. Research in xenograft mouse models has shown that miR-20a-5p confers resistance to anti-PD-1 therapy in TNBC [180]. In addition to regulating CD8 + T cell function to mediate resistance to anti-PD-1 therapy, several in vitro and in vivo experiments have indicated that exosomal circUHRF1 and circZNF451 induce immunosuppression in HCC and LUAD by impairing the activity of NK cells and macrophages, respectively, thereby promoting tumor immune evasion and resistance to anti-PD-1 immunotherapy [181–183]. Additionally, According to the study, analyses of human CRC immune profiles and tumor-immune cell interactions revealed that EVs containing miR-424 suppressed the CD28-CD80/86 costimulatory pathway in tumor-infiltrating T cells and dendritic cells, leading to immune checkpoint blockade resistance. Modified EV-miR-424 knocked down enhanced T-cell-mediated antitumor immune responses in CRC models and increased the immune checkpoint blockade response. Furthermore, intravenous injections of these modified EVs induced tumor antigen-specific immune responses and boosted the efficacy of immune checkpoint blockade in CRC models mimicking aggressively progressing, late-stage disease [84].

Taken together, these findings collectively suggest that EV-ncRNAs connect immune cells, creating a microenvironment that promotes tumor progression and malignancy. Additionally, they influence the efficacy of checkpoint blockade. Therefore, identifying and targeting key EV-ncRNAs involved in these interactions may reverse resistance to anti-PD-1 immunotherapy and significantly enhance the efficacy of checkpoint blockade.It is plausible that the use of the Aethlon ADAPT™ system to clear EV-ncRNAs from cancers may increase the effectiveness of anti-PD-1 immunotherapy [183].

### Strategies to target EV-ncRNAs.

As mentioned above, due to the pivotal role of EV-ncRNAs in cancer, developing effective inhibition strategies for EV-ncRNAs, such as antagonists, locked nucleic acids, or antisense oligonucleotides (ASOs), has become a formidable challenge. In recent years, various strategies have been employed to assess the therapeutic potential of EV-ncRNAs, specifically utilizing ASOs and RNA interference (RNAi) methods to counteract immunosuppressive activities. (Fig. 3B). For example, researchers from the City University of Hong Kong successfully utilized red blood cell-derived extracellular vesicles (RBCEVs) as carriers to deliver small interfering RNAs (siRNAs) and ASOs to leukemia and BC cells, significantly inhibiting cell proliferation by suppressing miR-125b expression. Intriguingly, RBCEVs have also demonstrated effective ASO delivery in in vivo experiments [184].

In addition to ASOs and RNAi, small molecule inhibitors can be utilized to target specific pathways or interactions involved in the biogenesis or function of these ncRNAs. According to the literature,it has been envisioned that small molecules could alter miRNA expression and their function in cells at the pre-transcriptional level. By modifying miRNA promoter regions, these molecules are thought to indirectly regulate miRNA expression [185]. For instance, miR-127, which targets the proto-oncogene BCL6 and is embedded in a CpG island, is known to be subject to epigenetic silencing in cancer cells [186–189]. The CRISPR/Cas9 system has been recognized as a revolutionary genome editing tool in diverse areas of molecular biology [190]. In the context of lncRNA research, the Cas9 nuclease can be utilized to delete lncRNA genes or introduce RNA-destabilizing elements into their locus [190]. Additionally, the nuclease-deficient dCas9 mutant, which maintains its RNA-dependent DNA-binding activity, can modulate gene expression when fused to transcriptional repressor or activator domains [191–193] (Fig. 3B). EV-miR-424 were found to suppress the CD28-CD80/86 costimulatory pathway in tumor-infiltrating T cells and dendritic cells in human CRC, leading to resistance to immune checkpoint blockade. Modified EV-miR-424 knocked down enhanced T-cell-mediated antitumor immune responses and increased the efficacy of immune checkpoint blockade in CRC tumor models. Furthermore, intravenous injections of these modified EVs induced tumor antigen-specific immune responses and boosted the immune checkpoint blockade efficacy in models mimicking aggressively progressing, late-stage disease [84]. Notably, the development of nanoparticle-based delivery systems has shown promise in effectively targeting and delivering inhibitory molecules to EV-ncRNAs within the tumor microenvironment [194].

In summary, these approaches present a multitude of instruments for altering the expression strategies of EV-ncRNAs, ultimately bolstering antitumor reactions and advancing their utilization in the context of cancer therapeutics.

## Conclusions and perspectives.

The emerging role of EV-ncRNAs in modulating the PD-1/PD-L1 pathway offers new insights into cancer immunotherapy and its clinical applications. The complex interactions among EV-ncRNAs, immune checkpoints, and the tumor microenvironment highlight the enormous potential of EV-ncRNAs as key targets in cancer treatment strategies. In this review, we re-examine the mechanisms by which EV-ncRNAs influence PD-1/PD-L1 expression across various cancers. Furthermore, we emphasize the crucial role of EV-ncRNAs in regulating immune cell dysfunction and drug resistance in cancer immunotherapy, highlighting the clinical application prospects of EV-ncRNAs as prognostic and diagnostic biomarkers in anti-PD-1/PD-L1 cancer immunotherapy. Despite the promising outlook, this field faces numerous challenges.

Firstly, does targeting the PD-1/PD-L1 pathway via EV-ncRNAs hold greater significance in cancer compared to other immune checkpoints, such as CTLA-4, LAG-3, and TIM-3? Currently, ICIs like PD1 and PD-L1 monoclonal antibodies have shown remarkable efficacy in cancer treatment, but therapies targeting single checkpoints often encounter issues such as drug resistance and limited efficacy [195]. Therefore, exploring the design of EV-ncRNAs that can simultaneously target multiple immune checkpoints may provide a potential solution to overcome the limitations of single-target therapies [196, 197]. Additionally, given the limitations of using ICIs alone, researchers are exploring combination therapies that integrate EV-ncRNAs with other treatment modalities. For example, combining EV-ncRNA-targeting therapies with anti-PD-1/PD-L1 immunotherapy aims to overcome tumor drug resistance and enhance treatment efficacy through synergistic effects across multiple targets and pathways [198]. In mouse models, the combination of exosomal circ_001264 siRNA with anti-PD-L1 significantly reduced leukemia tumor burden [163]. Moreover, exosomal miR-431-5p enhanced the efficacy of anti-PD1 therapy, particularly when combined with galunisertib, demonstrating synergistic inhibition of GC progression in C57BL/6 mice [199].

Secondly, as natural drug delivery vehicles, EV-ncRNAs exhibit excellent biocompatibility, low immunogenicity, and high targeting efficiency [200, 201]. How can we enhance the drug-loading capacity and stability of EV-ncRNAs and achieve precise targeted delivery? Engineering EV-ncRNAs can optimize their drug-loading capacity and stability. For instance, strategies such as altering EV membrane composition, introducing targeting molecules, or fusion proteins can improve the targeting specificity and delivery efficiency of EV-ncRNAs [202, 203]. Furthermore, while the advantages of EVs make them ideal for drug delivery, EV-based drug delivery still faces challenges, such as the lack of standardized isolation methods and limited clinical-grade production. However, more promisingly, a series of characterization and validation methods have been developed by many scientists for research and clinical applications to assess the purity of EVs and quantify their cargo. These methods include transmission electron microscopy, scanning electron microscopy, atomic force microscopy, nanoparticle tracking analysis, dynamic light scattering, resistive pulse sensing, enzyme-linked immunosorbent assay, flow cytometry, fluorescence-activated cell sorting, and microfluidics and electrochemical biosensors [204].

Thirdly, the potential off-target effects of targeted therapy against EV-ncRNAs in a clinical setting represent an urgent issue to address. Since EV-ncRNAs play roles in multiple cell types and physiological and pathological processes, targeted therapy against specific EV-ncRNAs may lead to unintended biological effects [205]. For example, certain ncRNAs may be expressed in different types of cells and tissues, and targeted drugs against these ncRNAs may interfere with the physiological functions of normal cells. Additionally, as natural nanocarriers, EVs can cross biological barriers and deliver carried ncRNAs to distant tissues, further increasing the risk of off-target effects. To address these issues, researchers are continuously exploring new isolation and characterization technologies, as well as developing more precise targeted therapy strategies. For example, optimizing EVs isolation conditions and improving RNA extraction efficiency can enhance the isolation and characterization of EV-ncRNAs [206, 207]. Additionally, in vitro studies have revealed that miRNA sponges, despite their ability to inhibit specific miRNAs and downregulate associated downstream targets, necessitate markedly higher concentrations (considerably higher than those for ASO-based inhibitors) to attain effective target mRNA inhibition, which may result in elevated unwanted off-target effects [208]. To overcome the technical impediments for effective drug delivery, chemical modifications have been designed for a newer generation of molecular mimics, with the goal of optimizing RNA oligonucleotide stability while limiting potential off-target effects [205].Meanwhile, utilizing gene editing technologies like CRISPR/Cas9 can precisely regulate the expression and function of ncRNAs, thereby reducing the risk of off-target effects [209, 210]. Lastly, considering the dual role of EV-ncRNAs in modulating PD-1/PD-L1 expression, future research should aim to mitigate the immunosuppressive effects of TEV-ncRNAs while harnessing their potential for immunostimulatory activation.

In summary, the ability of EV-derived ncRNAs to modulate the PD-1/PD-L1 pathway offers new strategies for enhancing the efficacy of cancer immunotherapy. By targeting EV-ncRNAs, it may be possible to restore immune function and overcome resistance to current therapies. For tumor immunologists, delving into the role of TEV-ncRNAs in immune checkpoint pathways remains a top priority, potentially providing new perspectives for cancer treatment.

## Data Availability

No datasets were generated or analysed during the current study.

## Abbreviations

EVs: Extracellular vesicles
NcRNA: Noncoding RNA
MiRNA: MicroRNA
LncRNA: Long noncoding RNA
CircRNAs: Circular RNA
UTR: Untranslated region
MREs: MiRNA response elements
MVB: Multivesicular bodie
ILV: Intraluminal vesicle
PC: Pancreatic cancer
VISTA: V-domain immunoglobulin suppressor of T-cell activation
PD-1H: Programmed death-1 homolog
HCC: Hepatocellular carcinoma
CRC: Colorectal cancer
TIME: Tumor immune microenvironment
dMMR: DNA mismatch repair deficiency
MSI-H: Microsatellite instability
TNBC: Triple-negative breast cancer
AdMSCs: Adipose mesenchymal stem cells
ICP: Immune checkpoint protein
MDSCs: Myeloid-derived suppressor cells
NSCLC: Non-small cell lung cancer
RCC: Renal cell carcinoma
ICIs: Immune checkpoint inhibitors
GC: Gastric cancer
LECs: Lymphatic endothelial cells
ER: Endoplasmic reticulum
BC: Breast cancer
TAM: Tumor-associated macrophage
ICC: Intrahepatic cholangiocarcinoma
ASOs: Antisense oligonucleotides
RNAi: RNA interference
RBCEVs: Red blood cell-derived extracellular vesicles
